# Emotional mimicry and smiling behaviors in schizophrenia: An ecological approach

**DOI:** 10.1038/s41537-025-00632-y

**Published:** 2025-06-07

**Authors:** Mathilde Parisi, Stéphane Raffard, Tifenn Fauviaux, Victor Vattier, Dorra Mrabet, Delphine Capdevielle, Ludovic Marin

**Affiliations:** 1 EuroMov Digital Health in Motion, Univ Montpellier, IMT Mines Ales, Montpellier, France; 2 Univ Paul Valéry Montpellier 3, EPSYLON EA, 4556 Montpellier, France; 3 University Department of Adult Psychiatry, CHU Montpellier, Montpellier, France

**Keywords:** Schizophrenia, Psychosis

## Abstract

Individuals with schizophrenia often experience social skill deficits, leading to reduced social interaction quality. Emotional mimicry, the automatic imitation of a counterpart’s expression, plays a crucial role in social interactions. This study introduces a novel methodology for assessing positive emotional mimicry during a naturalistic conversation. We recruited interacting partners (*n* = 20), each engaging in two interactions: one with an individual diagnosed with schizophrenia (*n* = 20) and one with a matched healthy control (*n* = 20). Participants were video recorded while taking turns sharing happy personal memories during six minutes. Using OpenFace, we detected participants’ emotional expressions and computed mimicry scores based on their temporal alignment. Consistent with our hypotheses, individuals with schizophrenia exhibited reduced smiling and positive emotion mimicry. Furthermore, interacting partners reported lower willingness to continue interacting with individuals with schizophrenia compared to healthy controls. This study stands out for its innovative methodology, assessing a key social skill in an ecological setting. Our findings highlight the potential of emotional mimicry training as an important intervention to improve social interaction in schizophrenia.

## Introduction

Navigating the social landscape is challenging for everyone. Yet, for individuals with a diagnosis of schizophrenia (SCZ), these everyday social interactions can feel like navigating an entirely different world, where the rules are elusive and the cues are often missed. Social dysfunction, which reflects impairments in “a person’s ability to interact appropriately and effectively in the social world”^1^, is a primary criterion of SCZ according to the DSM^2^. Social dysfunction in SCZ comprises poor social interactions, difficulties in maintaining relationships with family and friends, or performing effectively in the workplace^3–5^.

Social functioning relies on social skills, defined as “specific behavioral components or abilities that we need in order to communicate effectively or to be successful in social situations”^1^. Individuals with SCZ frequently exhibit social skills deficits. For instance, they often display altered nonverbal behaviors (e.g., reduced facial expressiveness), which is linked to lower social interaction quality^6,7^. A crucial social skill is emotional mimicry, which is the behavioral tendency to imitate a counterpart’s emotional expression during social interactions^8,9^. According to Hess and Fischer (2022), emotional mimicry is directly linked to the quality of social interactions; in fact, displaying affiliative emotional mimicry helps create a warm and pleasant atmosphere. In contrast, reduced or antagonistic mimicry is linked to a decline in the quality of social interactions^10^.

Various studies have assessed emotional mimicry in individuals with a diagnosis of SCZ, and the results are quite dispersed^11^. Two studies found no differences in emotional mimicry between healthy controls and individuals with a diagnosis of SCZ^12,13^, while four studies highlighted that individuals with a diagnosis of SCZ exhibit reduced emotional mimicry and/ or incongruent mimicry compared to healthy controls^14–17^. Notably, five of these studies measured participants’ mimicry in reaction to pictures or video clips of actors portraying facial expressions. Authors often favor this approach due to the methodological challenges of measuring emotional mimicry in naturalistic settings. However, recent research increasingly highlights the importance of preserving human social behavior’s inherently active, embodied, and reciprocal nature by employing experimental paradigms based on real-life, naturalistic stimuli^18^. This is particularly relevant in the context of SCZ, where social interaction difficulties are a core feature. Thus, assessing emotional mimicry in ecological settings is essential to fully capture the dynamics of interpersonal exchanges and their influence on the quality of social interactions^11^. Indeed, previous studies have shown that healthy individuals often report lower-quality social interactions when engaging with individuals with SCZ, compared to interactions with other healthy individuals^7,12,19^. These perceptions may be linked to disruptions in emotional mimicry, which plays a crucial role in shaping social connection and interaction quality^9^.

## Present study

This study aimed to assess positive emotional mimicry using an interactive procedure involving individuals with SCZ and healthy controls, employing a novel methodology. We recruited interacting partners (IP) to interact once with an individual with a diagnosis of schizophrenia (SCZ) and once with a healthy age and sex-matched participant (MAT). During the interactions, participants were seated across from each other and asked to share happy memories^12,20^. Each participant’s face was recorded using a camera. Using OpenFace, we first detected the positive facial expressions of each participant, which enabled the calculation of mimicry scores. The mimicry scores of the SCZ participants were compared with those of the MAT participants. In addition, we assessed the IP’s willingness to continue interacting with their conversation partners (i.e., SCZ and MAT), serving as an indicator of social interaction quality. Based on previous studies highlighting blunted affect^21^, reduced emotional mimicry^14–17^, and poorer social interaction quality^7,12,19^, we predicted that:

(H1): Individuals with a diagnosis of SCZ would exhibit reduced positive facial expressions compared to matched healthy controls.

(H2): Individuals with a diagnosis of SCZ would display reduced mimicry of positive facial expressions compared to matched healthy controls.

(H3): Interacting partners would report a lower willingness to continue interaction with individuals diagnosed with SCZ compared to matched healthy controls.

Finally, we aimed to test the correlation between mimicry scores and willingness to continue the interaction, as well as between mimicry scores and both SCZ symptoms and medication use.

## Transparency and openness

The study and the hypotheses were preregistered on the Open Science Framework (https://osf.io/huqnb). Analytic code and statistical analysis details can be found in the following repository (https://github.com/parisim/mimicry).

## Methods

This study is part of a larger project (Enhancer 2022-2026) investigating speech and gesture during social interactions in individuals with SCZ. Participants engaged in various conversational tasks and completed a series of questionnaires.

This protocol was ethically approved by the French ethical comity - Comité de la Protection des Personnes (2024-A00553-44). All participants received a written information letter and signed a consent form before the experiment. The French ethical comity approved the compensation for healthy participants, who received 50 euros for their participation. We report how we determined our sample size, all data exclusions, all manipulations, and all measures in the study.

### Participants

To determine the appropriate sample size, we conducted an ANOVA-based power analysis using MorePower 6.0^22^ for a between-subjects design with three groups—interacting partners (IP), individuals diagnosed with SCZ, and age- and sex-matched healthy controls (MAT)—a small effect size of f = 0.15, a power of 80%, and an alpha level of 0.05. The results of the power analysis indicated that a total of 60 participants (i.e., 20 per group) were required.

We recruited 60 participants in total, including 20 individuals meeting DSM-V criteria for SCZ (*M* = 32.12 years, SD = 9.47; 5 women, 15 men), 20 age- and sex-matched healthy controls (MAT, *M* = 30.80 years, SD = 12.00; 5 women, 15 men), and 20 interacting partners (IP, M = 26.40 years, SD = 6.54; 5 women, 15 men). A Mann-Whitney U test with continuity correction confirmed that the SCZ and MAT groups did not differ significantly in age (U = 158.5, *p* = 0.27), ensuring proper matching.

All control participants (i.e., IP and MAT) were adults residing in Montpellier, France, and were recruited via local associations. They had no personal history of psychosis or neurological or psychiatric disorders and were not taking medications that could affect cognition. Additionally, any control participants meeting clinical criteria for a major depressive episode or an anxiety disorder—assessed using the Mini-International Neuropsychiatric Interview^23^—were excluded. They also had no first-degree relatives with severe mental illnesses such as SCZ or bipolar disorder.

SCZ participants were recruited from the University Department of Adult Psychiatry in Montpellier. At the time of the study, all were in a stable phase of their illness and receiving antipsychotic treatment. Symptom severity was assessed using the Positive and Negative Syndrome Scale (PANSS^24^), and antipsychotic doses were converted into chlorpromazine (CPZ) equivalents (see Table 1). We used the Montreal Cognitive Assessment (MOCA)^25^ to control cognitive differences, the Beck Depression Inventory (BDI-II)^26^ to control major symptoms of depression, and the National Adult Reading Test to control for premorbid intelligence (fNart)^27^. No differences were found for these items (*p* > 0.05) except for MoCA (*p* = 0.03).

**Table 1:** Participants descriptive.

	IP (*n* = 20)		MAT (*n* = 20)		SCZ (*n* = 20)		MAT vs. SCZ	
	M	SD	M	SD	M	SD	Statistics	*p*-value
Age	26.40	6.54	30.80	12.0	32.15	9.47	*U* = 158.5	*p* = 0.27
fNART	29.1	3.85	28.63	5.30	26.74	7.48	*U* = 213.5	*p* = 0.34
MoCA	28.79	1.13	28.68	1.89	26.68	3.3	*U* = 254.5	*p* = 0.03
BDI-II	8.25	4.52	9.6	9.47	11.84	8.73	*U* = 143.5	*p* = 0.29
PANSS Positive					12.72	5.33		
PANSS Negative					18.17	11.49		
PANSS General					35.56	18.03		
CPZ equivalent dose in mg					305.05	262.39		
Illness Duration (years)					7.75	4.57		
Gender (M/W)	15/5		15/5		15/5			

*IP* Interacting partner, *MAT* healthy subjects matched in age and sex, *SCZ* individuals with a diagnosis of schizophrenia, *MoCA* Montreal Cognitive Assessment, *BDI-II* Beck Depression Inventory-II, *PANSS* Positive and Negative Symptom Scale, *CPZ* Chlorpromazine.

### Questionnaire

The Willingness To Interact Scale^28^ is a six-item instrument designed to assess a participant’s willingness to engage in various hypothetical social situations with a specific interacting partner (e.g., sitting next to the person during a 3-h bus ride). Participants responded using a Likert scale from 1 “absolutely not” to 5 “absolutely”. Additionally, we have included three supplementary items that measure the participant’s willingness to get to know the person better, their level of liking, and their perceived similarity to the conversation partner.

### Procedure

The IP completed the experiment twice, once with the SCZ and once with the MAT. The order of the interactions was counterbalanced between participants. The IP had never met with the SCZ or the MAT before and was unaware of the SCZ diagnosis. Upon arrival at the hospital, the two conversation partners were welcomed and introduced to each other in a room equipped with two chairs and two cameras. In this room, they received information letters and signed consent forms.

Participants were seated 1.5 m apart, facing each other, with a camera positioned behind them to capture their conversation partner. Before the interaction, the experimenter recorded a baseline of the participants’ facial expressions. Participants introduced themselves naturally during this recording while maintaining a neutral facial expression. Following the baseline recording, participants completed four conversational tasks as part of the Enhancer project: an icebreaker for initial introductions, a free conversation, a structured conversation, and a final conversation, which is the focus of analysis in this study. During this final conversation, participants were asked to share happy memories for approximately three minutes per participant. Previous face-to-face studies have used this approach of narrating an emotional memory to assess emotional mimicry^12,20^. During the interaction, the dyad was alone in the room to avoid being disturbed by the experimenter. The experimenter used a beep to signal when three minutes had passed, prompting participants to switch narrating roles, and concluded the interaction after six minutes. After the interaction, participants were seated at separate desks and asked to complete the Willingness to Interact Scale^28^. After completing the first interaction, the IP took a short break before repeating the process with their second conversation partner (i.e., the SCZ or the MAT).

### Data analysis

Our novel methodological approach is grounded in the theoretical definition of emotional mimicry as outlined by Hess and Fischer (2013, 2014). According to their model, emotional mimicry is characterized by four key features: (1) both individuals display matching emotional expressions, (2) these expressions are temporally aligned, occurring within seconds of each other, (3) the mimicker’s expression is contingent upon the other person’s expression, and (4) mimicry reflects a sharing of the original emotional display, rather than just a reactive response^9^. Building on these principles, our assessment of emotional mimicry involved several steps: First, we identified positive emotional expressions from both participants. Next, we ensured temporal alignment by focusing on shared smiles, which are defined as instances where both participants smiled within two seconds of each other. Finally, we calculated emotional mimicry for each participant. To carry out this analysis, we used OpenFace 2.2, an open-source software that monitors facial action unit activity according to the Facial Action Coding System^29^. Our methodological approach offers several advantages: First, unlike electromyography, OpenFace does not require the use of electrodes on participants’ faces, thus avoiding potential intrusiveness and the risk of signaling to participants that their facial expressions are being measured. Second, we focus on detecting specific facial expressions rather than assessing synchrony across the entire interaction, which could be biased by verbal communication.

#### Action units’ analysis

Using OpenFace 2.2, we extracted the facial action unit (i.e., AU, specific movement of the face) activation in both the baseline recording (i.e., 10 s) and the happy memory segment (i.e., 6 min). Following established guidelines for assessing positive facial expressions^30–32^, we focused on three action units activation: AU4 (brow furrow, associated with corrugator supercilii activity), AU6 (cheek raiser, associated with orbicularis oculi activity), and AU12 (lip corner puller, associated with zygomaticus major activity).

To accurately assess participants’ emotional mimicry based on these facial action unit activations, it is essential to perform a baseline correction that accounts for each participant’s natural expressivity. Consequently, action unit activations during the happy memory recording were z-standardized within subjects using the baseline recording for the three previously mentioned action units (i.e., AU04, AU06, and AU12). The within-subject z-standardization was performed for each action unit as follows: zAU = (AU during the emotional video – mean AU of the baseline video) / standard deviation of AU during the emotional video. This standardization method allows the baseline trial to serve as the “zero point,” with deviations from this baseline indicating relative activation or deactivation of the facial action units^33^. As a result, values not significantly different from zero suggest no meaningful changes in action unit activation. In contrast, positive or negative values indicate a relative increase or decrease in action unit activation during the trial. This baseline correction approach has been previously employed in studies measuring emotional mimicry^33^.

Following the baseline correction, we computed a score of positive emotional expression: ((AU12 + AU06)/2) – AU04, following previous studies’ guidelines^30–32^. We used this formula because, as previously emphasized, a positive facial expression is indicated by increased activity of the muscles responsible for smiling (i.e., indexed by AU12 and AU6) and decreased activity of the muscle involved in frowning (i.e., indexed by AU4). Our focus on comparing such scores of positive emotional expressions, rather than the activation of specific action units, aligns with the definition of emotional mimicry by Hess and Fischer (2013, 2014). They propose that emotional mimicry doesn’t require an exact replication of the displayed emotion but rather involves matching the emotional expressions based on their valence, which is captured by the score of positive emotional expression^8,9,34^. From this point onward, the positive facial expressions detected will be referred to as ‘smiles.’ However, it is important to note that these ‘smiles’ were identified through analysis of the entire face, as a smile is not solely determined by mouth movement but also by eye wrinkling and reduced frowning.

By the end of this step, we had a continuous signal of the baseline-corrected score of positive facial expressions for each participant during the 6-min interaction. The signal was recorded at 30 Hz, with one score assigned per frame. We applied a second-order low-pass filter with a cut-off frequency of 0.05.

#### Smile detection

The second step involved detecting peaks of positive facial expressions or smiles. As previously emphasized, our goal was to investigate mimicry based on the detected facial expressions rather than synchrony scores across the signals of the entire interaction, in line with Hess and Fischer’s (2013, 2014) definition of emotional mimicry. Following the methodology of Lavelle et al.^19^ for hand movement detection, we defined a positive expression (i.e., a smile) as any instance where the positive emotional expression signal exceeded the threshold of one standard deviation above the participant’s mean score^19^.

For each peak in the signal surpassing this threshold, we recorded the frame number corresponding to the start of the smile (i.e., the first frame above the threshold) and the end of the smile (i.e., the last frame above the threshold). When two smiles were detected within less than 2 s of each other, they were merged using the start of the first smile and the end of the second. Two independent researchers, blinded to the participant’s diagnosis, then visually verified the smile detection using OpenFace 2.2, which displayed a picture for each frame with a visual overlay around the participant’s face. Based on visual verification, some corrections were necessary. Consistent with previous studies using automatic detection, we found that automatic detection never fully eliminates the need for human verification^35^. Of all the automatically detected smiles, 8.6% were removed after visual verification, primarily due to facial obstructions. Additionally, 7.8% of smiles were manually added to supplement the automatic detections. For manually added smiles, independent researchers identified the first and last frames in which participants displayed a smile. Disagreements were solved by seeking consensus.

At the end of this step, we recorded the total number of smiles produced by each participant, along with the frame numbers marking the start and end of each smile. Using these frame numbers, we calculated the total duration each participant spent smiling during the interaction, which we referred to as the ‘smile ratio.’

#### Shared smile detection

**Fig. 1:**
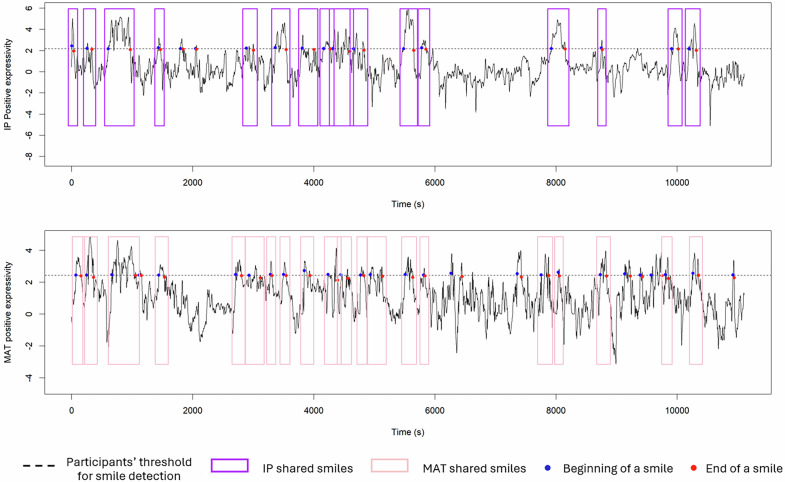
Shared smile detection. *Note:* The upper graph displays the facial activity signal of one IP participant, while the lower graph illustrates the facial activity of the corresponding MAT participant. The x-axis represents time in seconds, while the y-axis displays the positive emotional expression score (baseline-corrected and within-subject z-standardized). The dotted lines indicate each participant’s smile detection threshold, calculated as the mean score plus one standard deviation across the entire recording. Blue dots mark the onset of detected smiles, while red dots denote their offset. Violet rectangles highlight the smiles of the IP shared with those of the MAT, and pink rectangles represent the smiles of the MAT shared with those of the IP.

After the smile detection and visual verification, we identified instances when both participants smiled simultaneously (i.e., a shared smile^36^,). Following Hess and Fischer’s (2014) recommendations, emotional mimicry typically occurs within one second of stimulus onset. However, as previous research has shown that the same smile is detected approximately 800 ms later by automatic facial recognition than electromyography^37^, we extended the time window to two seconds. Thus, we defined shared smiles as those occurring within a two-second interval where both participants were smiling. For each participant, we assessed the number of smiles shared with at least one smile from another participant. As a result, two conversation partners may have a different number of shared smiles. For example, two smiles from the IP could overlap with a single smile from the MAT. In this case, the IP would have two shared smiles, while the MAT would have only one (see Fig. 1).

#### Emotional mimicry scores

Finally, we calculated emotional mimicry scores. For this step, we considered the number of smiles each participant had and the number of smiles shared between them. The mimicry score for each participant was determined by dividing the number of shared smiles by the total number of smiles produced by the other participant. For example, in the graph shown in Fig. 1, the IP displayed 18 smiles, 16 of which were shared with the MAT. Therefore, the MAT’s mimicry score is the ratio of shared smiles (16) to the total number of smiles produced by the IP (18), resulting in a score of 0.89. Similarly, the IP mimicry score is calculated by dividing the number of smiles produced by the MAT shared with the IP (20) by the total number of smiles produced by the MAT (27), resulting in a score of 0.74. As previously noted, the number of shared smiles can vary between conversation partners, as one person’s smile may overlap with multiple smiles from the other participant, as seen in this example.

## Results

Before testing our hypotheses, we confirmed that participants (i.e., SCZ, MAT, and IP) did not differ in baseline activation of the action units. We conducted a linear mixed model analysis for each action unit of interest during the baseline recording, specifically AU04, AU06, and AU12. Group membership (MAT, SCZ, or IP) was included as a fixed effect, and participant ID was modeled as a random intercept. Tukey’s HSD correction was applied to adjust for multiple comparisons. The results revealed no significant effect of group on any of the AUs examined, indicating that baseline facial expressions did not differ across groups (AU04: MAT vs. SCZ: β = −0.13, *p* = 0.51; IP vs. SCZ: β = −0.27, *p* = 0.06; AU06: MAT vs. SCZ: β = −0.16, *p* = 0.39; IP vs. SCZ: β = −0.14, *p* = 0.51; AU12: MAT vs. SCZ: β = 0.03, *p* = 0.97; IP vs. SCZ: β = −0.14, *p* = 0.58).

### (H1): Individuals with a diagnosis of schizophrenia would exhibit reduced positive facial expression compared to matched healthy controls

**Fig. 2:**
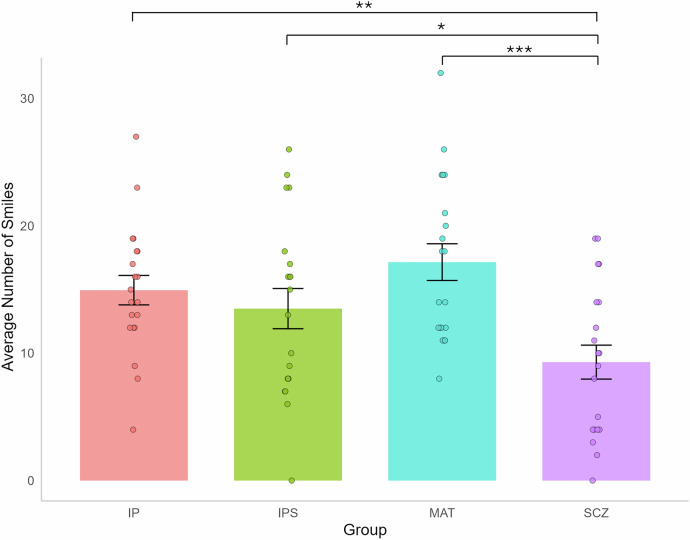
Number of smiles for each group. *Note:* IP: interacting partner conversing with MAT controls. IPS: interacting partner conversing with SCZ participants. MAT Healthy controls matched in age and sex with SCZ participants. SCZ Individuals diagnosed with schizophrenia. Significance level: *(*p* < 0.05), **(*p* < 0.01), ***(*p* < 0.001). Error bars represent the standard error of the mean (SEM).

We conducted a mixed linear model analysis on the number of smiles, with the group (i.e., IP, MAT, SCZ) as a fixed factor and participant ID (i.e., identifier) as a random intercept to account for individual differences and repeated measures (i.e., IP when interacting with the SCZ participant and with the MAT participant). Tukey HSD corrections were applied to control the familywise error rate. The results indicated that SCZ participants exhibited significantly fewer smiles than MAT controls (MAT vs. SCZ: β = 7.85, *p* < 0.0001, CI95[3.70, 12.00], Cohen’s d = 1.58). Additionally, we compared the number of smiles produced by SCZ participants to those of IP in both conditions. We refer to IP as ‘IPS’ when interacting with SCZ participants and as ‘IP’ when interacting with MAT. The results indicate that SCZ participants displayed significantly fewer smiles than IPS (IPS vs. SCZ: β = 4.20, *p* = 0.046, CI95[0.05, 8.35], Cohen’s *d* = 0.85) and fewer smiles than IP (IP vs. SCZ: β = 5.65, *p* = 0.0036, CI95[1.50, 9.80], Cohen’s d = 1.14). In contrast, no significant difference emerged between the IP, the IPS, or the MAT (IP vs. IPS: β = 1.45, *p* = 0.79, CI95[−2.70, 5.60]; IP vs. MAT: β = -2.20, *p* = 0.50, CI95[−6.35, 1.95]; IPS vs. MAT: β = -3.65, *p* = 0.10, CI95[−7.79, 0.50], see Fig. 2).

**Fig. 3:**
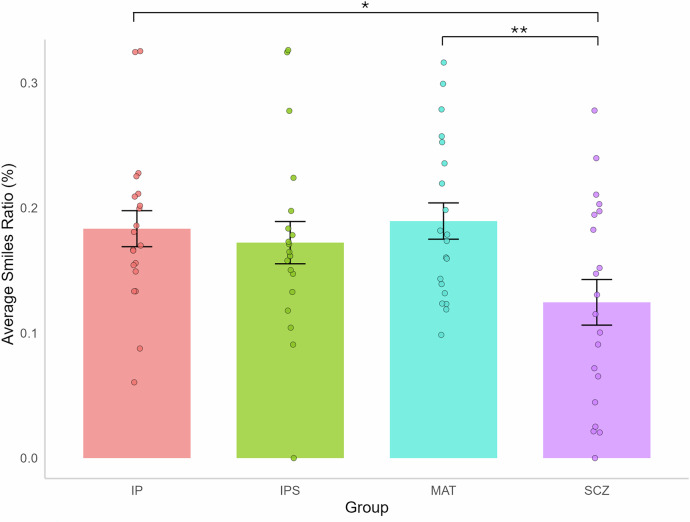
Smile ratio for each group. *Note:* IP: interacting partner conversing with MAT controls. IPS: interacting partner conversing with SCZ participants. MAT: Healthy controls matched in age and sex with SCZ participants. SCZ Individuals diagnosed with schizophrenia. Smile ratio The percentage of the interaction during which a participant smiles. Significance level: *(*p* < 0.05), **(*p* < 0.01), ***(*p* < 0.001). Error bars represent the standard error of the mean (SEM).

To further examine smiling behavior, we conducted an additional analysis of the smile ratio (i.e., the proportion of time each participant spent smiling during the interaction). We performed a linear mixed model analysis on the smile ratio, with the group (i.e., IP, MAT, SCZ) as a fixed factor and participant ID as a random factor to account for individual differences and repeated measures (i.e., IP vs. IPS). Tukey HSD corrections were again applied to control the familywise error rate. The results showed that SCZ participants spent significantly less time smiling than MAT controls (MAT vs. SCZ: β = 0.06, *p* = 0.0078, CI95[0.01, 0.12], Cohen’s *d* = 1.06). Similarly, SCZ participants smiled less than IP (IP vs. SCZ: β = 0.06, *p* = 0.0189, CI95[0.007, 0.11], Cohen’s *d* = 0.96). Despite a trend effect, no significant difference emerged in the smile ratio between the SCZ and the IPS (IPS vs. SCZ: β = 0.05, *p* = 0.0785, CI95[−0.004, 0.10]). Additionally, and similarly to the number of smiles, no difference emerged in the smile ratio between the IPS, the IP, and the MAT (IP vs. IPS: β = 0.01, *p* = 0.94, CI95[−0.04, 0.06]; IP vs. MAT: β = −0.006, *p* = 0.99, CI95[−0.06, 0.045]; IPS vs. MAT: β = −0.02, *p* = 0.81, CI95[−0.07, 0.034], see Fig. 3).

Consequently, SCZ participants smiled less frequently and for a shorter duration during the interaction compared to MAT controls. Additionally, no significant difference was found in the interacting partner’s smiling behavior when conversing with SCZ versus MAT participants (i.e., IPS vs. IP) in terms of both the number of smiles and the smile ratio. These findings suggest that interaction partners displayed similar smiling behaviors regardless of their conversation partners.

### (H2): Individuals with a diagnosis of schizophrenia would display reduced mimicry of positive facial expressions compared to matched healthy controls

**Fig. 4:**
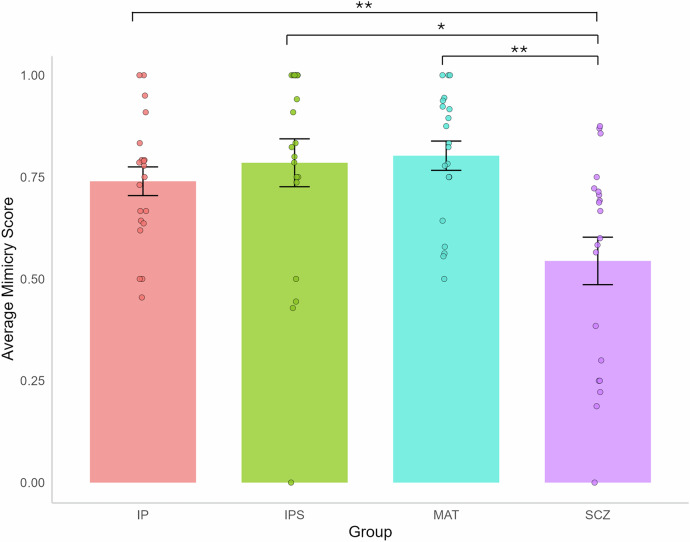
Mimicry scores for each group. *Note:* IP: interacting partner conversing with MAT controls. IPS: interacting partner conversing with SCZ participants. MAT Healthy controls matched in age and sex with SCZ participants. SCZ Individuals diagnosed with schizophrenia. The mimicry score for each participant was determined by dividing the number of shared smiles by the total number of smiles produced by the other participant. Significance level: *(*p* < 0.05), **(*p* < 0.01), ***(*p* < 0.001). Error bars represent the standard error of the mean (SEM).

We conducted a mixed linear model analysis on the mimicry score, with the group (i.e., IP, MAT, SCZ) as a fixed factor and participant ID as a random factor to account for individual differences and repeated measures (i.e., IP vs. IPS). Tukey HSD corrections were applied to control the familywise error rate. The results indicated that SCZ participants exhibited less mimicry of the IP compared to MAT controls (MAT vs. SCZ: β = 0.26, *p* = 0.0015, CI95[0.08, 0.43], Cohen’s d = 1.23). Additionally, SCZ participants displayed significantly less mimicry than IPS (IPS vs. SCZ: β = 0.24, *p* = 0.0034, CI95[0.06, 0.42], Cohen’s d = 1.15) and less mimicry than IP (IP vs. SCZ: β = 0.20, *p* = 0.024, CI95[0.02, 0.37], Cohen’s d = 0.93). In contrast, no significant difference emerged between the IP, the IPS, or the MAT (IP vs. IPS: β = −0.05, *p* = 0.90, CI95[−0.22, 0.13]; IP vs. MAT: β = −0.06, *p* = 0.78, CI95[−0.24, 0.11]; IPS vs. MAT: β = −0.02, *p* = 0.99, CI95[−0.19, 0.16]) (see Fig. 4).

Consequently, SCZ participants showed reduced emotional mimicry compared to MAT controls. In addition, no significant difference was found in the interacting partner’s mimicry behaviors when conversing with SCZ versus MAT participants (i.e., IPS vs. IP). These findings suggest that interaction partners displayed similar mimicry behaviors regardless of their conversation partners.

### (H3): Interacting partners would report a lower willingness to continue interaction with individuals diagnosed with schizophrenia compared to healthy controls

We conducted a linear mixed model analysis on the willingness total score (sum of the score of the nine items) with the group (reference: MAT) as the fixed effect and a random intercept for the dyad number. We applied Tukey HSD correction to control for the familywise error rate. Results indicated a significant effect of the group on the willingness to interact (MAT vs. SCZ: β = 6.5, *p* = 0.022, CI95[1.37, 11.63], Cohen’s *d* = 1.76), highlighting that the IP expressed diminished willingness to continue the interaction with the SCZ compared to the MAT. We conducted separate analyses for each item of the willingness, and significant results were found for the following items: take advice from (MAT vs. SCZ: β = 0.80, *p* = 0.01, CI95[0.19, 1.41], Cohen’s d = 1.81), sit next to on the bus (MAT vs. SCZ: β = 1.00, *p* = 0.001, CI95[0.39, 1.61], Cohen’s *d* = 2.27), invite to home (MAT vs. SCZ: β = 0.65, *p* = 0.038, CI95[0.036, 1.26], Cohen’s d = 1.47), get married to relative (MAT vs. SCZ: β = 0.70, *p* = 0.026, CI95[0.086, 1.31], Cohen’s *d* = 1.59), work with (MAT vs. SCZ: β = 0.95, *p* = 0.002, CI95[0.34, 1.56], Cohen’s *d* = 2.15), liking (MAT vs. SCZ: β = 0.65, *p* = 0.04, CI95[0.04, 1.27], Cohen’s *d* = 1.47), and similarity (MAT vs. SCZ: β = 0.75, *p* = 0.02, CI95[0.14, 1.36], Cohen’s *d* = 1.70). The results indicate that IP participants showed a reduced willingness to continue interacting with SCZ participants compared to MAT controls, as reflected in the total willingness score and all items’ scores.

Furthermore, to assess the perceived quality of the interaction for MAT and SCZ participants as well, we conducted an additional analysis comparing their ratings of willingness to continue interacting with the IP. We conducted a linear mixed model analysis on the willingness total score (sum of the score of the nine items) with the group (reference: IP) as the fixed effect and a random intercept for the dyad number. We applied Tukey HSD correction to control for the familywise error rate. Results indicated a significant effect of the group on the willingness to interact (IP vs. IPS: β = -5.15, *p* = 0.006, CI95[−8.39, 1.91], Cohen’s *d* = 0.98). In other words, SCZ participants expressed significantly lower overall willingness to continue interacting with the IP compared to MAT participants.

### Correlations

We first examined whether mimicry scores or smiling behaviors correlated with the willingness to continue interacting. However, the correlations were not significant for mimicry scores (*r* = 0.05, *p* = 0.84), the number of smiles (*r* = 0.07, *p* = 0.76), or the smile ratio (*r* = 0.02, *p* = 0.92), indicating no meaningful association between SCZ participants’ mimicry and smiling behaviors and the IP’s willingness to continue the interaction.

We conducted additional analyses to examine whether mimicry behaviors correlated with SCZ participants’ symptoms and medication dosage, as measured by chlorpromazine equivalents. Given that we tested five correlations (i.e., medication, PANSS Positive, PANSS Negative, PANSS General, and PANSS Total scores), we applied a Bonferroni correction and adjusted the significance threshold to *p* < .01. The results revealed a significant negative correlation between mimicry scores and negative symptoms assessed by the PANSS (*r* = −0.48, *p* = 0.046). Furthermore, significant negative correlations were found between mimicry scores and both the total PANSS score (*r* = −0.48, *p* = 0.045) and the general psychopathology score of the PANSS (*r* = −0.57, *p* = 0.015). These results suggest that increased negative symptoms and general symptoms of psychopathology, as well as overall symptoms of SCZ, were associated with reduced emotional mimicry. However, none of these correlations remained significant after applying the Bonferroni correction.

## Discussion

This study aimed to assess emotional mimicry in individuals with SCZ using a novel methodology that captures this variable in a natural setting. Consistent with our first hypothesis, we found that individuals with SCZ smiled significantly less frequently and for shorter durations than matched healthy controls (H1). Additionally, aligning with our second hypothesis, individuals with SCZ exhibited reduced emotional mimicry compared to matched healthy controls (H2). Finally, individuals with SCZ received more negative ratings from their conversation partners regarding their willingness to continue interacting, compared to matched healthy controls, thus confirming our third hypothesis (H3).

We initially hypothesized that individuals with SCZ would exhibit reduced positive facial expressions compared to healthy controls, given that blunted affect is a core negative symptom of SCZ^21,38^. Blunted affect specifically reflects a deficit in emotional expression—particularly in facial displays of emotion—rather than in the experience of emotion itself^38–40^. Numerous studies have consistently demonstrated reduced facial expressiveness in individuals with SCZ compared to healthy controls^21,41–43^. Our findings align with this body of research, confirming that individuals with SCZ exhibit diminished facial expression relative to healthy controls. Yet, our study stands out, as few have used automated methods like OpenFace to assess facial expressions in SCZ^44^, especially in an ecological setting involving natural interactions between individuals with SCZ and healthy participants.

Our second hypothesis predicted that individuals with SCZ would exhibit reduced emotional mimicry compared to healthy controls. Supporting this, we found that individuals with SCZ mimicked their conversation partners significantly less than healthy controls. These findings align with four previous studies that examined emotional mimicry in SCZ using images or video clips of actors displaying emotional expressions^14–17^ and with one study that assessed facial mimicry during a silent board game^45^. However, our results contrast with those of Riehle and Lincoln (2018), the only other study to have assessed emotional mimicry during a conversation between individuals with SCZ and healthy controls.

Both our study and Riehle and Lincoln (2018) used a similar procedure to induce mimicry—asking participants to narrate an emotional memory^12,20^. However, our methods for measuring emotional mimicry differed significantly. While Riehle and Lincoln (2018) assessed the synchrony of facial activity throughout the entire interaction using cross-correlation analysis, we focused on the alignment of detected facial expressions within the theoretical framework of Hess and Fischer (2013, 2014, 2022). This methodological difference may explain the discrepancy in results, as verbal communication can activate the zygomaticus muscle (responsible for smiling), potentially affecting cross-correlation scores. Interestingly, their study also found no difference in smiling behavior between individuals with SCZ and healthy controls, contrasting with previous research^21,41–43^. These results further confirm that analyzing overall muscle activity during conversation, rather than identifying specific facial expressions, may explain the failure to detect reduced smiling and mimicry activity in individuals with SCZ. Future research should replicate our findings and methodology, potentially applying them to Riehle and Lincoln’s (2018) dataset to better understand these differences and confirm whether they stem from methodological choices.

Similarities appear to emerge in the explanations for reduced smiling and emotional mimicry in individuals with SCZ. While blunted affect in SCZ is not due to diminished emotional experience^46^, its underlying mechanisms remain debated. Three predominant explanations suggest that blunted affect results from a complex interplay of altered brain activation patterns, social skill deficits, and potential medication effects^47,48^. In this study, no medication effects were found, ruling out this factor as the primary explanation. Instead, our findings reveal a significant correlation between emotional mimicry and negative symptoms, pointing to a potential link with social skill deficits. Cowan et al.^49^ argue that these deficits play a crucial role, suggesting that reduced social motivation in individuals with SCZ contributes to blunted affect. Unlike healthy controls, individuals with SCZ may not use positive emotional expressions as signals of affiliation but rather express emotions only when genuinely felt^49^. This aligns with prior research showing that individuals with SCZ exhibit a reduced willingness for affiliation compared to healthy controls^50,51^. This reduced affiliative motivation may also explain their diminished emotional mimicry. Indeed, Hess & Fischer (2013, 2014, 2022) argue that affiliation stance is the key antecedent of emotional mimicry. A possible mechanism underlying this reduced affiliative motivation is impaired reward learning. Individuals with SCZ often struggle to reciprocate social interactions appropriately and adjust their behavior based on feedback, potentially due to difficulties in processing positive social reinforcement^49,52^. Cowan et al.^49^ emphasize that deficits in learning from positive outcomes may hinder social success over time, ultimately limiting the ability to recognize the benefits of affiliation and affiliative behaviors, such as emotional mimicry. This diminished affiliative drive also echoes what Minkowski described as the *trouble générateur* of SCZ—namely, autism^53^. According to Minkowski, SCZ is fundamentally marked by a loss of “vital contact with reality,” which impairs an individual’s capacity to resonate with the world and empathize with others^54^. Much like autism, SCZ can thus be understood as a condition affecting intersubjectivity, characterized by a reduced bodily resonance with others. This attenuation in embodied connection may contribute to difficulties in social engagement, including emotional mimicry^55^.

Consistent with our third hypothesis, our study found that interacting partners reported a reduced willingness to continue engaging with individuals with SCZ compared to healthy controls. These findings align with previous research^12,19^, further highlighting the diminished quality of social interactions in individuals with SCZ. While our study does not establish a direct link between emotional mimicry or smiling behaviors and willingness to continue interacting, the lower quality of social interactions in SCZ may be attributed to a broader range of social skill deficits. For instance, other behavioral impairments may include disorganized speech^56^ or averted gaze^57^, which may contribute, along with emotional mimicry deficits and blunted affect, to reduced social interaction quality. Individuals with SCZ also reported significantly lower willingness to continue interacting with their interaction partner compared to matched controls, further supporting the notion of reduced interaction quality in this population. This finding aligns with numerous studies indicating diminished affiliative motivation in individuals with SCZ^50,51^, as well as evidence that they tend to engage in lower-quality social interactions and report more negative emotions following such interactions^58^.

The negative consequences of reduced emotional mimicry and blunted affect extend beyond diminished social interaction quality. In SCZ, impaired social interactions are associated with a range of adverse outcomes, including reduced quality of life and increased mortality rates^59,60^. Blunted affect has also been linked to an increased risk of suicide^38^, lower quality of life, depressive symptoms, impaired social functioning, emotional withdrawal, and negative self-evaluation^61^. Thus, addressing these deficits is crucial for improving long-term outcomes in individuals with SCZ.

Enhancing emotional mimicry in individuals with SCZ may significantly improve social interaction quality, given its strong association with various social benefits^8–10,34^. However, Hess and Fischer (2013, 2014, 2022) emphasize that while emotional mimicry serves as a social interaction tool and is linked to personality traits (Parisi et al., in press), it differs from other abilities because it occurs automatically and outside conscious awareness, making it impossible to learn directly. Consequently, they argue that the only way to enhance emotional mimicry is by increasing its primary antecedent, willingness for affiliation. Psychosocial approaches, such as social skills training, have improved appropriate expressivity^62^, alongside Cognitive Behavioral Therapy^63^. Additionally, a recent meta-analysis suggests that oxytocin may have positive effects on social cognition^64^. However, the effectiveness of both pharmacological and psychosocial interventions in addressing blunted affect in SCZ remains limited to negligible^65^, highlighting the need for novel or more targeted therapeutic strategies. Psychosocial interventions should also aim to enhance emotional mimicry, and their effectiveness should be systematically evaluated.

Our study has several limitations that should be addressed in future research. To begin, we assessed only the emotional mimicry of positive emotions. Future studies should also examine negative emotions, as different patterns may emerge. Additionally, future research could also further investigate whether reduced positive emotional mimicry in individuals with SCZ reflects a general reduction in expression or the presence of divergent emotional responses. In this context, future studies could incorporate alternative measures of blunted affect, such as the Clinical Assessment Interview for Negative Symptoms^66^ or the Scale for the Assessment of Negative Symptoms (SANS)^67^, to examine whether these assessments are associated with reduced smile detection and emotional mimicry. Another limitation concerns our smile detection methodology. While we introduced a new methodology for measuring emotional mimicry in ecological settings, emphasizing the importance of analyzing detected emotional expressions within the theoretical framework of Hess and Fischer (2013, 2014, 2022), our smile detection technique, which relies on movement detection^19^, has room for improvement, as indicated by a false positive detection rate of 8.6%. Though automated methods have been extensively validated against electromyography and manual coding^68–70^, they remain less precise and are subject to temporal delays compared to electromyography^37,71^. Previous studies emphasize that automated techniques can never fully eliminate the need for visual verification^35^. While we acknowledge that automatic detection software is not the most precise tool for smile detection, we believe it was the best choice for our study. Its use in this clinical population allowed for a naturalistic setting by avoiding placing electrodes on participants’ faces, helping maintain a calm demeanor, and closely replicating real social interactions. Furthermore, while previous studies have shown that OpenFace is more precise than other automatic facial action unit detection tools such as FaceReader and Py-Feat^72,73^, and is also free and open-source, other software like AFFDEX also appears promising^71^. Future studies could aim to replicate our methodology using these alternative tools to compare their effectiveness. Additionally, to reduce the false positive detection rate, future studies could also consider combining multiple methodologies, such as incorporating human coding. Finally, we did not analyze participants’ speech in terms of quantity, valence, or content, nor did we examine potential differences in mimicry between speaking and listening phases. These factors could have influenced the results, as meta-analyses have identified impaired autobiographical memory as a major cognitive deficit in individuals with SCZ^74^.

## Data Availability

Data and analyses are available in the following repository: https://github.com/parisim/mimicry.
